# Smart Lighting Clinical Testbed Pilot Study on Circadian Phase Advancement

**DOI:** 10.1109/JTEHM.2019.2937957

**Published:** 2019-08-30

**Authors:** Joseph D. Gleason, Meeko Oishi, Michelle Simkulet, Arunas Tuzikas, John P. Hanifin, George C. Brainard, S. R. J. Brueck, Robert F. Karlicek, Lee K. Brown

**Affiliations:** 1 Electrical and Computer EngineeringUniversity of New Mexico 1104 AlbuquerqueNM87131USA; 2 Lighting Enabled Systems and Applications Engineering Research CenterRensselaer Polytechnic Institute 8024 TroyNY12189USA; 3 Department of NeurologyThomas Jefferson University 6559 PhiladelphiaPA19107USA; 4 Internal MedicineUniversity of New Mexico 1104 AlbuquerqueNM87131USA

**Keywords:** Lighting control, system implementation, circadian rhythm, biomedical engineering

## Abstract

Objective: Lighting is a strong synchronizer for circadian rhythms, which in turn drives a wide range of biological functions. The objective of our work is a) to construct a clinical in-patient testbed with smartİ lighting, and b) evaluate its feasibility for use in future clinical studies. Methods: A feedback capable, variable spectrum lighting system was installed at the University of New Mexico Hospital. The system consists of variable spectrum lighting troffers, color sensors, occupancy sensors, and computing and communication infrastructure. We conducted a pilot study to demonstrate proof of principle, that 1) this new technology is capable of providing continuous lighting and sensing in an active clinical environment, 2) subject recruitment and retention is feasible for round-the-clock, multi-day studies, and 3) current techniques for circadian regulation can be deployed in this unique testbed. Unlike light box studies, only troffer-based lighting was used, and both lighting intensity and spectral content were varied. Results: The hardware and software functioned seamlessly to gather biometric data and provide the desired lighting. Salivary samples that measure dim-light melatonin onset showed phase advancement for all three subjects. Conclusion: We executed a five-day circadian rhythm study that varied intensity, spectrum, and timing of lighting as proof-of-concept or future clinical studies with troffer-based, variable spectrum lighting. Clinical Impact: The ability to perform circadian rhythm experiments in more realistic environments that do not overly constrain the subject is important for translating lighting research into practice, as well as for further research on the health impacts of lighting.

## Graphical abstract

We present a clinical in-patient testbed with feedback-capable, variable-spectrum smart lighting and evaluate its feasibility for use in future clinical studies. A five-day circadian rhythm study that varied intensity, spectrum, and timing of lighting as proof-of-concept for future clinical studies with troffer-based, variable spectrum lighting demonstrated the ability to perform circadian rhythm experiments in more realistic environments that do not overly constrain the subject. These results are important for translating lighting research into practice, as well as for further research on the health impacts of lighting.

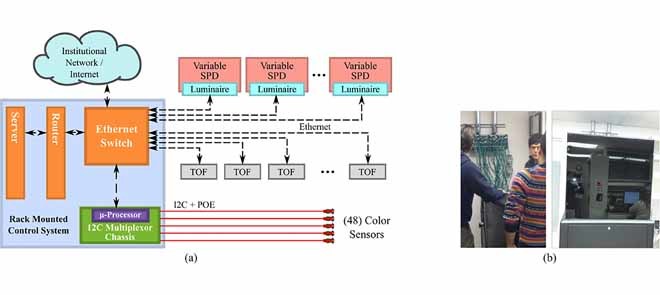

## I. Introduction

Advances in light emitting diode (LED) technology have made tunable spectral combinations commercially feasible, with a growing market for tunable lighting (i.e., Signify (formerly Phillips Lighting), Acuity Brands) and lighting control software (i.e., Nest, F.lux, Philips Hue). “Smart” lighting, that incorporates feedback from personal sensing devices (actigraphy, skin temperature, and others) into the control of variable spectrum LED light fixtures, has the potential for enormous impact in a variety of environments, including classrooms, workplaces, health care facilities, and many other arenas [1]–[4]. In hospitals, there is emerging evidence that carefully designed lighting (that varies intensity as well as spectral power distribution) may improve recovery times and even health outcomes [4]–[6]. Further, light exposure has been shown to affect not only circadian phase, but also attention, cognitive throughput, and alertness [7]–[10].

Lighting is a strong synchronizer for circadian rhythms, a 24-hour periodic cycle in biological processes that drives a wide range of biological functions. Circadian disruption has been associated with serious health effects, including gastrointestinal disorders, diabetes, obesity, cardiovascular disorders, and an increased risk of cancer [11]–[15]. Delayed sleep-wake phase disorder, which affects more than one million Americans (many of whom are teenagers), is associated with poor school performance and mood disturbances [1], [16]. Further, a higher likelihood of accidents is prevalent for the 20% of American workers with non-standard hours [13], [17]. Many of these and other findings have fueled concerns about the ubiquity of electrical lighting.

Smart lighting systems also have the potential to improve health and well being. In hospitals, smart lighting could be used to re-entrain circadian rhythms in intensive care and post-operative patients. Variable spectrum lighting has been installed in health care facilities in Scandinavia [3], [18] and in the United States [19]; these facilities consist of full spectrum, color tunable lighting fixtures. Lighting in workplaces and in homes could help night-shift workers and others with non-traditional hours. Worker productivity and energy conservation are the focus of research at the Well Living Lab [20], which uses correlated color temperature (CCT) tunable fixtures within a restricted variable CCT range. At Rensselaer Polytechnic Institute (RPI), a variable spectrum lighting system was installed in the Smart Conference Room [21], with the support of the National Science Foundation (NSF) Engineering Research Center in Lighting Enabled Systems and Applications (LESA). The Smart Conference Room has been employed to investigate energy conservation through sensing of external lighting [21], integration of occupancy sensing and activity detection with lighting controls [22], [23], and lighting to improve cognitive throughput [24], amongst others.

**FIGURE 1.**
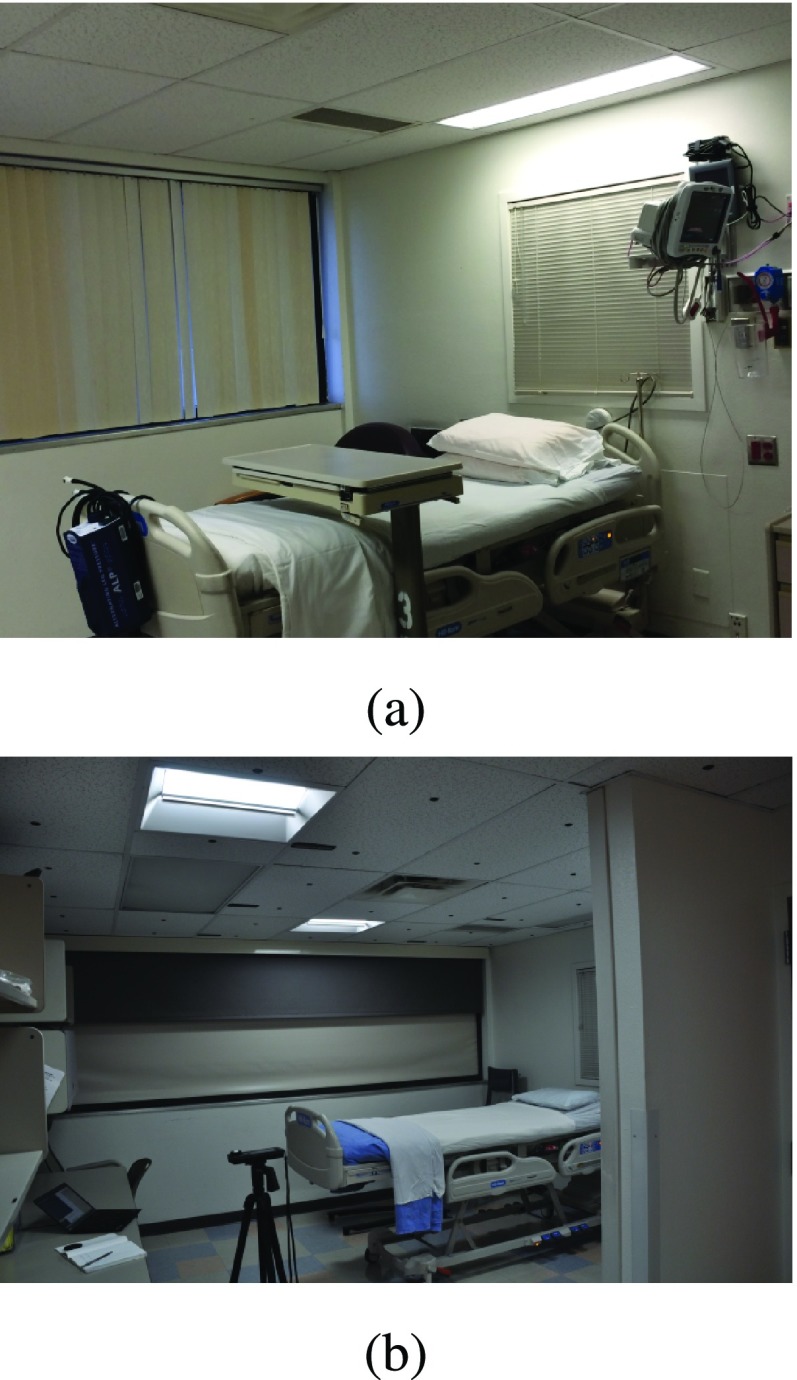
The UNM smart lighting clinical testbed (a) prior to installation and (b) after installation.

LESA recently helped facilitate construction of an in-patient, smart lighting clinical testbed at the University of New Mexico (UNM) Hospital (Figure 1). The testbed is located in the Clinical and Translational Science Center (CTSC), which is part of an NIH-funded consortium for translation of fundamental science into technology for patient care. The UNM smart lighting clinical testbed is a unique facility in the US: it is an integrated system that consists of full spectrum LED troffers, time-of-flight sensors for occupancy detection, and color sensors. In contrast to other deployed variable spectrum lighting systems, the additional sensing capabilities enables the potential for real-time, biometric feedback in lighting control. Further, because the system is internet-based, the testbed can also be coupled with internet-based wearable devices. The testbed allows a wide variation in spectral power density, and the capability of customization and adaptation to individual subjects. Additionally, the choice of software architecture and device management facilitates multi-day experiments under controlled conditions, a duration necessary for experiments on the time-scale of circadian processes.

A preliminary version of this work has been reported in [25], which focused on the testbed hardware and installation. Here, we describe the results of a small, pilot study conducted in the testbed, demonstrating its feasibility for use in future clinical studies, and also describe the design of reliable and robust software and middleware, which was critical for operation of the testbed over many consecutive days. The remainder of the paper is as follows: Section II describes the testbed design (including hardware, middleware, and software) and installation. Section III presents the experiment design for the pilot study, which focused on circadian phase advancement. Results of the pilot study are presented in Section IV and discussed in Section V. Conclusions are presented in Section VI.

## II. Background and Approach

### A. Infrastructure Elements

**FIGURE 2.**
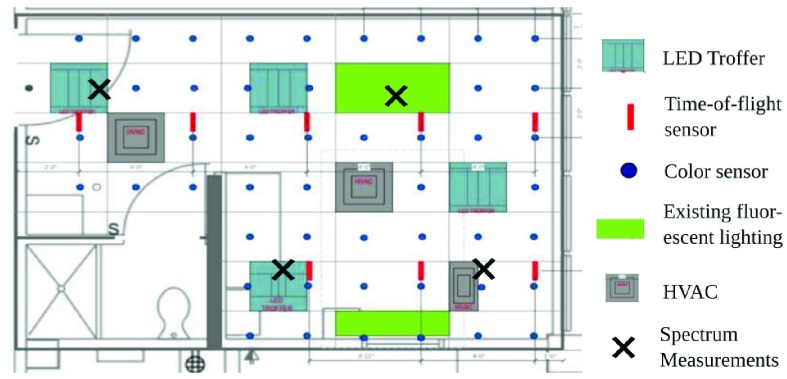
Physical layout of testbed infrastructure.

The UNM Smart Lighting Clinical Testbed is based on a design previously implemented at RPI in the Smart Conference Room [21]. Most elements of the RPI design are duplicated, with some significant differences due to constraints arising from a clinical environment. The system (Figure 2) consists of 4 Telelumen Penta Luminaires [26], 8 Iris IRMA MATRIX time-of-flight sensors [27], and 48 AMS TCS34725 color sensors [28].

The Telelumen Penta Luminaire fixtures are controllable, variable spectrum lighting sources consisting of five LED channels: red, green, blue, and phosphor-converted amber and white. The intensity of each channel can be varied individually, allowing for a broad selection of colors and intensities. Empirically, the luminaires can generate white light in the range of 1800–10000 K, with a color rendition index (CRI) greater than 66. Each color-tunable light fixture is connected, via ethernet, to an ethernet switch, to enable communication to each source independently.

**FIGURE 3.**
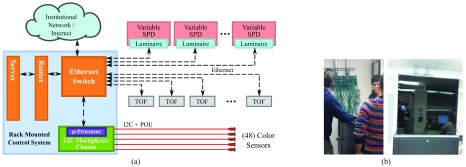
(a) Network schematic to control variable spectrum luminaires. (b) Server without (left) and with (right) customized plexiglass covering.

The color sensors, manufactured by AMS-TAOS, are small, low-voltage, 4-channel RGBC sensors measuring red, green, blue, and clear (unfiltered) color intensities. Each sensor communicates via an I2C interface and is powered over ethernet. The sensors are connected to six, 8-channel multiplexors, which connect to a Raspberry Pi microcontroller (Figure 3). The microcontroller has custom code for receiving data from the color sensors, and is connected to the local network to allow communication with the rest of the testbed system.

The IRMA MATRIX time-of-flight devices each consist of an array of $25\times 20$ single-pixel time-of-flight sensors. Each pixel time-of-flight sensor measures the approximate distance from the ceiling to an object underneath. The pixel time-of-flight sensors are aligned at different angles of incidence from the ceiling, to provide a broad field of view. The combined pixel sensors allow for a privacy preserving measurement of objects or persons in the room. The time-of-flight sensors are intentionally coarse to assure privacy, and can be polled as frequently as 10 Hz.

The IRMA MATRIX devices communicate through a proprietary messaging protocol that uses the Universal Datagram Protocol (UDP). Thus, communication with the time-of-flight sensors must be implemented in C++, via the manufacturer’s (Iris-Gmbh) Application Programming Interface (API). Recent development in sensor technology has enabled small, inexpensive, time-of-flight sensors that have open communication protocols, such as the Heptagon OLIVIA [29].

### B. System Design

Two key design restrictions associated with the clinical environment were 1) the need for ongoing use of the room as a standard hospital room, with standard lighting, when not in use for research, and 2) the need for manual control of the sensors and actuators by the study coordinator or the charge nurse. To address the former need, the variable spectrum lighting system was installed alongside the existing lighting system (Figure 1(b)). Existing lighting fixtures and HVAC elements restricted placement of the sensors and actuators (Figure 2). Regarding the latter need, a hard switch to shut off power to the luminaires was installed, covered by a hinged box to discourage patient use but still allow the charge nurse to power the luminaires off, if necessary.

As shown in Figure 3(a), sensors and actuators are wired to an internet connected server. The server is located in an adjacent clinical laboratory that is operated by the CTSC. The laboratory maintains a College of American Pathologists certification, which requires that all surfaces must be regularly wiped down to prevent particulate contamination. Ethernet cabling that connects the devices to the remote server was fed through the ceiling of the testbed (Figure 3(b), left), then into a custom designed plexiglass covering for the server (Figure 3(b), right), for easy cleaning.

In an effort to avoid particulate contamination, physical access to the server room was significantly limited. Hence, establishing remote access to the server for troubleshooting, maintenance, and testing was paramount. The remote system access is protected by two layers of security: one in accessing the local intranet, and another through password secured user accounts. A Raritan power distribution unit was installed to allow remote disconnection of the power, in the event of unexpected device behavior, e.g. if the luminaires remained on even when the wall switch was turned off.

The devices in the testbed communicate via socket communication, which is supported by most programming languages, e.g. Python, C++, C#, MATLAB, etc.

### C. Software Design

Development of reliable middleware is of the utmost importance for implementation of code that must run continuously for at least five days, and for which in-person troubleshooting and testing is quite limited. We developed methodological approaches for data management and fault handling (including diagnostics, alerting, “soft” fails, consistent time-stamping, and other functionality), as well as a framework for code sharing (with history, version control, branching, tags, changes in ownership, etc.).

All of the developed code is stored in an online Git repository, to collaboratively share code between the Smart Conference Room at RPI and the Smart Lighting Clinical Testbed at UNM. The new repository and code sharing enabled developments in the Smart Lighting Clinical testbed to be applied to the Smart Conference Room, and vice versa.

The code for the clinical testbed was developed in Python, except for the code for the time-of-flight sensors, which was written in C++, due to device requirements. Python was selected for several reasons. First, it is open source, which eliminates licensing requirements and potential errors arising from license expiration. Second, it is an interpreted language, as opposed to a compiled language, which allows for fast prototyping. Because of the very limited testing and debugging time available, this was crucial. Finally, Python is a stable language with strong socket communication interfacing, allowing it to successfully run for up to five days.

**FIGURE 4.**
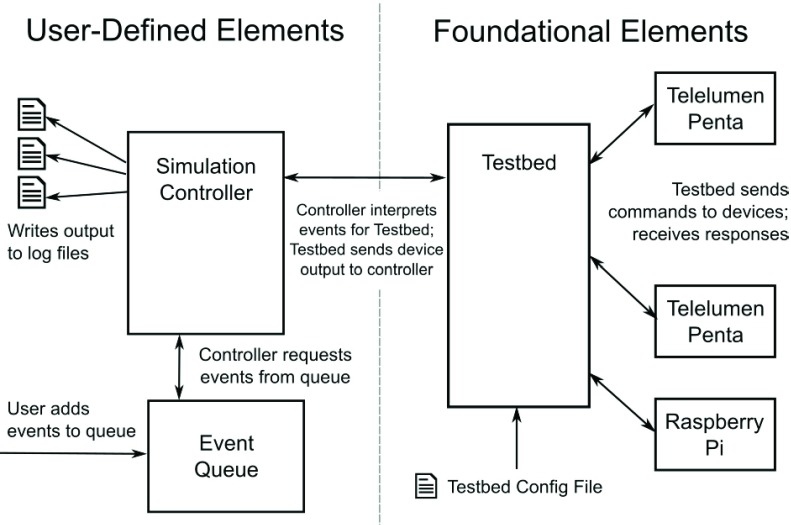
Software design schematic; foundational elements are consistent across testbeds with testbed-specific configuration files and an event-driven controller design so that researcher could design for multiple systems or create custom simulations.

The code is structured in two categories: foundational elements, which include class structures for controlling how to communicate with the luminaries and other sensors, and user-defined elements, which are specific to an experiment design (Figure 4). This separation was critical for designing software that was functional across multiple testbed environments. The foundational elements allowed for a consistent communication language to be developed such that individual researchers could easily create the commands necessary for a given experiment design. Each environment contained a testbed configuration file, which defined the network location and type of various elements in the testbed, allowing experiments to be developed that could be implemented in different testbeds.

To implement the desired lighting profiles for the pilot study, lighting commands needed to be absolutely timed, e.g. “turn lights to 1800 K at 18:30 on September 12, 2017.” To achieve this, two features were implemented: 1) a queuing system combining absolute date-time stamps and priority values (to resolve equal date-time conflicts), and 2) a simulation controller that would check for available events from the queue to execute. The simulation controller also checks the connections to the various testbed elements, e.g. the luminaires, to ensure that there are no connectivity problems.

Another challenge for implementation was the strict need for continuous execution over a duration of up to five days. In order to ensure that the testbed operated correctly without failure for the entire duration of the experiment, extensive debugging was performed on actions that could cause errors. Additionally, an error-reporting system was created to handle both soft and hard errors. The error-reporting system contacts an on-call administrator in the event of either soft or hard errors. Soft errors are errors that do not require that the simulation software be reset, such as a failure to communicate with the color sensors (which would not affect patient experience during the pilot study). A hard error, such as failure to connect to the Telelumen luminaires, requires the immediate action of the administrator to reset the system.

### D. System Installation and Validation

Installation of the variable spectrum lighting system required approval by and coordination amongst multiple entities, including NSF LESA, the UNM Center for High Technology Materials, the UNM Hospital, the UNM Sleep Disorders Center, and the CTSC. A core group of faculty who represented the research priorities for the completed testbed held discussions with these entities individually as well as jointly. A memorandum of understanding with the CTSC, that outlines responsibilities and anticipated usage of the room after installation, was completed. Because the UNM Hospital has extremely high usage (it is the only Level 1 trauma center in New Mexico), and is almost always near capacity, access to the room was typically determined on extremely short notice (usually after an occupant was discharged).

Our approach to design of the system was based on that implemented in the RPI Smart Conference Room. We first characterized the pre-existing light intensity: data were gathered with a light meter at two heights, 30 and 46 inches, at locations on a grid with 2 foot spacing in two directions, excluding the area occupied by the bed. The room was modeled in LitePro 2.0, and the light output of the luminaires predicted under several configurations. The selected configurations assure that light levels have at least the same value as compared to standard hospital lighting, and an acceptably uniform distribution of light at the two working heights. Uniformity is typically represented as the ratio of the minimum illuminance with respect to the maximum illuminance in a given area [30], and captures the evenness of the spatial distribution of light; values of 0.7 or higher are typically considered acceptable by the architectural and lighting design community. Lastly, the placement of the time-of-flight sensors was guided by the need for complete coverage with minimal overlap.

UNM Hospital Facilities requested bids for the installation from contractors familiar with the special requirements associated with a clinical environment (e.g., particulate containment and mitigation). Once a bid was selected, an installation date was scheduled around known patient requirements, with 1.5 days for installation and 0.5 days for troubleshooting. Due to the room’s frequent use, additional time for troubleshooting was scheduled opportunistically, based on room availability. Blackout shades were installed for full control of light in the room. The hardware installation was completed in November 2015, and all troubleshooting completed by April 2016.

## III. Methods and Procedures

Because the testbed is novel, and incorporates state-of-the-art technology for lighting, sensing, and control, that is atypical of existing testbeds, the pilot study presents a preliminary investigation to identify what changes would be necessary in preparation for future, full-scale clinical studies in the testbed. The primary goal of the pilot study is to demonstrate proof of concept, that is, to show that it is possible for the testbed to a) deliver color-tunable lighting, to sense occupancy, and to sense spectral content of the lighting sources, in an in-patient testbed over the duration of a multi-day study, without service interruptions or other technical failures, and b) to successfully recruit subjects who complete the full study. A secondary goal of the pilot study was to to determine if established techniques for circadian regulation and testing of lighting can be used in this unique testbed. We chose to focus on circadian regulation, since irregular and interrupted sleep can be common in hospital settings [31].

### A. Subject Eligibility

The experiment protocol was approved by the UNM Human Research Review Committee through Study ID 16-178 on July 21, 2016.

Eligibility criteria for the pilot study included subjects between the ages of 18–65 with no diagnosed sleep disorders. Subjects additionally were required to refrain from alcohol consumption for the duration of the hospital stay, as well as during the sleep screening for two weeks prior. Three females, with ages 33, 62, and 37, participated in the study.

### B. Experiment Design

During the two weeks prior to admission to the hospital testbed, each subject wore an ActiGraph wActiSleep-BT [32] actigraphy device, to monitor activity level. The actigraphy device was worn continuously, except when bathing. Additionally, each subject maintained a sleep diary to log sleep and wake times, that was updated each morning. Subjects were told to refrain from consumption of alcohol during this two-week monitoring phase, but were otherwise not restricted in their activities.

On the day of admission to the hospital testbed, each subject arrived midday. Subjects returned the actigraph device for data collection, and had blue-light filters [33] placed on any device that they intended to bring with them into the hospital room, including phones, computers, or tablets. The filter characteristics, as described by the manufacturer, are intended to block all wavelengths below 530 nm, and most wavelengths below 550 nm, i.e., those corresponding to blue light. The subject then returned to the hospital sometime 17:00–18:00, at which time the subject was admitted by the nursing staff. The subject was given a new actigraph to wear for the duration of the in-testbed portion of the study. Device model ActiGraph wActiSleep-BT was used for subjects 010 and 012, and an ActiGraph GTX3+ [34] was used for subject 006. The subject’s height, weight, and blood pressure were measured. During the subject’s stay in the testbed, all food consumption was strictly regulated, no alcohol consumption was allowed, and sleep-altering medications were not allowed.

In the testbed, the subject was allowed to move freely around the room and use electronic devices with applied filters (including the television, and the subject’s phones or computers). The experimental protocol required that:

• The subject was not allowed to leave the room, and the door to the hospital room remained closed, except for when nursing or hospital staff entered or exited the room.
• During dim-light melatonin onset (DLMO) measurements, the subject was not allowed to use any electronic devices and was instructed to avoid physical activity.
• During periods of darkness (sleep opportunity), the subject was not allowed to use any electronic devices.

To ensure that the subject did not use electronic devices during DLMO measurements and sleep periods, the devices were collected and stored in a locked container, accessible only to the nursing staff. The devices were returned after the DLMO measurements or sleep period ended.

Salivary melatonin measurements were collected twice during the subject’s stay, on days 1 and 4. Samples were collected every 30 minutes for a period of 6 hours on day 1 and 8 hours on day 4, for a total of 28 samples per subject.

Salivary samples followed the procedure in [35]. Samples were collected with Salivettes (cat. 51.1534; Sarstedt Australia Pty. Ltd., Mawson Lakes, South Australia) while the subjects lay in a supine position in bed, in dim light (<10 lux, 1800 K). Food and water were consumed only after saliva collection to reduce contamination or dilution of the sample. Participants were instructed to place the swab in their mouth and accumulate saliva for 2 min. After collection, samples were stored at −20 Celsius. For analysis, samples were thawed and centrifuged for 10 min at 2500 rpm, the swabs were removed from the casing, and the supernatant retained. A sensitive (4.3 pM) direct radioimmunoassay using reagents from Buhlmann Laboratories AG (Allschwil, Switzerland) was used to measure melatonin in the saliva.

### C. Lighting Protocol

**FIGURE 5.**
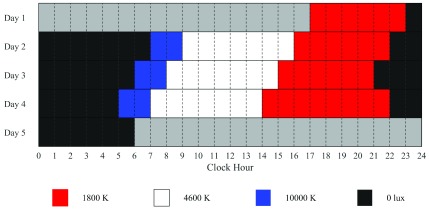
Lighting protocol for the UNMH pilot study for subject 006. Lighting was set to 3 pre-selected color temperatures, 1800 K, 4600 K, 10000 K, or was turned off. The gray bars represent times that are not part of the study. Lighting illuminances are shown in Table 1.

We designed a lighting protocol, shown in Figure 5, based on one used in light-box therapy [37], to phase advance subjects with troffer-based LED lighting. The protocol consisted of high color temperature light (10 000 K) in the mornings, standard illumination (4600 K) during the day, and a 6 hour period of dim, low light (1800 K) in the evening, before an 8 hour sleep period with no lighting (0 K). For comfort of the subject, there was a smooth 15 minute transition between each color temperature setting.

The starting sleep time for the study was determined via examination of the sleep diary kept for the two weeks prior to admittance to the testbed. The sleep hour was set one hour earlier each day to facilitate phase advancement. On the final day, an 8 hour dim light duration was used instead of the typical 6 hour duration to allow for more melatonin sampling. After melatonin sampling on the fourth day, the subject could either remain in the testbed to sleep the rest of the night or could be discharged immediately.

### D. Spectral Measurement

**FIGURE 6.**
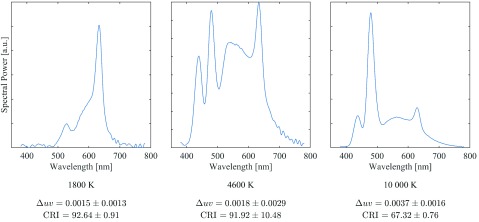
Spectral power of 1800 K, 4600 K, and 10 000 K light settings as measured by Sekonic Spectromaster C-7000 [36].

For each color temperature used in the study, spectral measurements were taken at four locations in the room, as indicated in Figure 2. At each location, five measurements were taken using a Sekonic Spectromaster c-7000: one facing the ceiling and one facing each primary wall of the room. For all measurements, the Spectromaster was raised 44 inches from the floor. Figure 6 shows a representative spectral power density for each color temperature, as well as the mean and standard deviations of the CRI and $\Delta uv$ values obtained from all measurements for each setting. The $\Delta uv$ values satisfy the ANSI white specification, i.e. $\Delta uv \leq 0.0054$ [38], [39].

The photometric and photopigment parameters are shown in Table 1. The photopigment parameters were computed from spectral power measurements using a freely available, web-based toolbox [40]. This toolbox was adopted in a CIE report and distributed internationally [41]. More recently, this toolbox has been developed into an international standard [42].

**TABLE 1:** Representative Photometric and Photopigment Parameters for the Three Color Temperatures Used in the Pilot Study

Radiometric and Photometric Values [380, 780] [nm]					
CCT			Photon Flux [cm/s^2^]	Irradiance [$\mu$W/cm^2^]	Photopic Illuminance [lux]
1800 K			$9.31 \times 10^{12} $	3.06	9.26
4600 K			$3.84 \times 10^{14}$	137	403
10000 K			$2.15 \times 10^{14}$	80	191.0
Retinal Photopigment Weighted IIluminances($\alpha$ -opic lux)					
CCT	S conc	Melanopsin ipRGc	Rod	M Conc	L Conc
1800 K	0.76	2.41	367	6.45	9.60
1000 K	276	354	367	389	398
10000 K	222	320	279	230	203

## IV. Results

During the approximately 15 days of operation for the pilot study, no hard errors occurred in the software. One soft error occurred while subject 006 was in the testbed; the color sensors refused a connection from the server due to an error made during system start-up.

The salivary samples obtained during the in-patient study were analyzed using the Burgess and Eastman 2005 method [44] for assessing DLMO. The DLMO threshold times and resulting phase differences are shown in Table 2. All three subjects experienced a phase advance in DLMO during the experiment.

**TABLE 2:** Dim-light Melatonin Onset Threshold Times Using the Burgess and Eastman 2005 Methods [43]. Negative Values of Phase Difference Indicate Phase Advancement

Burgess and Eastman Method			
	Day 1	Day 4	Difference [min]
006	21:44	20:06	−98
010	20:09	18:46	−83
012	19:59	19:06	−53

**FIGURE 7.**
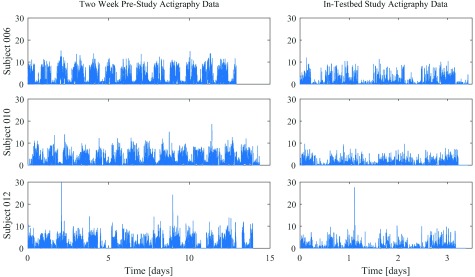
Actigraphy data for all three subjects, during the two weeks prior to the in-patient study (left), and during the in-testbed portion of the experiment (right).

The actigraphy data for each subject is shown in Figure 7.

## V. Discussion

### A. Testbed Capabilities

The pilot study successfully demonstrates the ability to conduct multi-day experiments in which lighting was varied in both intensity and spectrum, while simultaneously gathering other non-invasive data. Despite limited physical access for troubleshooting, the robustness built into the software helped assure success of the testbed in delivering the commanded lighting protocols and logging the sensed measurements. There were no faults in the physical or software design that would cause interruption or corruption of the data gathered during the study.

This testbed enables investigation of challenging research questions associated with more realistic lighting conditions and living environments. First, research into the design of spectral and intensity characteristics to affect human health is still in nascent stages. Many studies test light emitting devices that emit single spectrums of monochromatic, narrow bandwidth or broad bandwidth (e.g. fluorescent) light [7], [45], [46], not found in typical indoor lighting environments. A testbed that can vary both spectrum and intensity could facilitate research in how the spectral content as well as the dynamic nature of the spectral content can affect human health. This could have impact on research on disorders for which light-box therapy has been employed, such as traumatic brain injury [47], mood disorders [48], [49], Parkinson’s disease [50], and dementia [51].

Second, the spectral power distribution emitted from the luminaires is necessarily different than the spectral power distribution experienced by the subject. In conjunction with ongoing advances in wearable color sensors, the testbed’s capacity for active feedback of color and intensity sensing can help drive lighting systems to precisely deliver the necessary light.

Third, the rapid advances in wearable devices means that real-time, non-invasive data can be integrated with this testbed, to facilitate both information gathering (i.e., non-photic influences on circadian rhythms) as well as personalized lighting control. For example, continuously gathered actigraphy data has been used to non-invasively estimate a subject’s circadian phase in an ongoing manner [52]–[54], but this estimate could also be used to provide feedback-driven commands to the lighting system in an ongoing fashion.

### B. Circadian Phase Advancement

The circadian phase advancement observed in study participants could be the result several factors, including shifting the timing of sleep, shifting the timing of light exposure, as well as changing the spectral content and irradiance of the lighting. Unfortunately, isolating the effect of any of these is not possible, due to the nature of the pilot study. Further, the presence of device screens, albeit with blue-light filters, further confounds the ability to causally identify factors responsible for the phase shift. The phase advancement indicates that it is possible to implement a circadian-altering protocol in the testbed.

### C. Study Limitations

A significant limitation of the current study is the small sample size of the pilot group. Further studies would benefit from a larger cohort as well as using a more homogenous participant pool. Factors to control in future lighting comparison studies would include age, health status, including medication use, morningness/eveningness of their sleep cycle, weight, as well as managing or eliminating caffeine, alcohol and nicotine consumption. In addition, a study design using a parallel or crossover control group also experiencing an advanced timing lighting condition but where exposures remain fixed at a constant intensity and wavelength mixture as a comparison to variable spectrum and intensity protocol could potentially lead to more conclusive results.

Another limitation was the allowance of electronic, light-emitting devices, such as phones and computers, in the study area. While these devices can affect a person’s circadian phase, we opted to allow subject to use devices since for our population for recruitment, students and others affiliated with the university, we anticipated that banning devices for the duration of the experiment would make subject recruitment and retention extremely difficult, if not impossible.

Additionally, when using tunable, solid state (LED) lighting, we face trade-offs in achieving good visual performance, promoting beneficial health effects and limiting energy use [5], [6]. Specifically, in the pilot study reported here, the 10 000 K stimulus had a CRI of only 66, but the minimum CRI for good light quality is at least 80 [30]. For the LED devices used, to achieve a 10 000 K stimulus, CRI could not be raised to a minimum of 80. The solid state lighting system being installed on the International Space Station faced the same problem [55]. As LED lighting technology improves, achieving high CRI and high color temperature will likely be attainable. The new Telelumen Octa Luminaires report the ability to generate high CRI lighting up to 90 000 K [26].

## VI. Conclusions

The testbed facility constructed at the University of New Mexico Hospital is the only active clinical testbed designed for circadian research that is equipped with variable spectrum and intensity lighting technologies, occupancy sensing, and color spectra sensing. A pilot study was conducted to demonstrate the operation of the testbed and implementation of established protocols (based on light-box technology) for circadian regulation. The capabilities associated with this testbed could facilitate research and development of customized, feedback-based, spectrum-variable lighting to improve health, safety, and productivity, in controlled lighting environments.
